# 

**Published:** 1917-01-27

**Authors:** 


### An Old Journal with a New Name.

Commencing with the January issue, 1917, the *Louisville Monthly Journal of Medicine and Surgery* will become the official organ of the Mississippi Valley Medical Association, appearing with that issue in new dress and under the name of the *Mississippi Valley Medical Journal*. The Proceedings of this Association have been for some years of the highest scientific nature, and the appearance in full of the papers and discussions in convenient form for binding will enable members and subscribers to preserve these valuable Proceedings. The journal will have an epitome department. A full list of editors and contributors is added.

### Medical Appointments.

DR. CHARLOTTE BROWN has been appointed temporary assistant tuberculosis officer by the Essex County Council, at a salary of £350 per annum, with travelling expenses.

MISS MARGARET KATHLEEN MINET has been appointed superintendent of the Day and Night Nursery of the Willesden Council at a salary of £2 a week, wtih meals.

DR. TITTERTON has resigned his position as Assistant Medical Officer at Clare Hill Sanatorium, and his resignation as part-time Tuberculosis Officer under the Middlesex County Council necessarily follows. The Local Government Board has sanctioned the appointment of Dr. Chate as part-time Tuberculosis Officer. Dr. Chate was formerly in the service of the County Council as Medical Inspector of School Children.

EAST HAM CORPORATION HEALTH COMMITTEE recommends the appointment of Miss K. A. Bower, Hampstead, and Mrs. Ada A. Woodman, of the Infirmary, Caerleon, near Newport, Mon., as Health Visitors at a salary of £100 per annum, rising by annual increments of £10 per annum to a maximum annual salary of £120.

RATES OF SUBSCRIPTION: Twelve months. 6/6; Six months, 4/-; Three months, 2/-; post free to any address in the United Kingdom. Abroad, Twelve months, 8/8; Six months, 5/-; Three months, 2/6, post free. THE HOSPITAL "Index" to Volume LX., April to September 1916, is now ready, price 2½d. per copy, post free. Address The Manager, "THE HOSPITAL," The Hospital Building, 28/29 Southampton Street, Strand, London, W.C.

### HUMAN TEMPERAMENTS:

### Studies in Character.

By Dr. CHARLES MERCIER.

**1/3 net. By Post 1/4.**

"The essays are written in Dr. Mercier's well-known style—they are witty, pithy, and display a wide range of observation and learning. We recommend the brochure cordially to our readers. It is always extremely readable and often very wise."—*The Lancet.*

"Dr. Mercier is a well-known authority on mental diseases, and his practical knowledge assists him in this analysis of the temperaments of the artist, the faddist, the practical man, the envious man, the philosopher, and so on. Dr. Mercier can claim that the reader may find some widening of his knowledge of human nature as the result of these shrewd and experienced studies."—*The Times.*

"It is long since within so small a compass so much help was given to mankind in the pursuit of that 'proper study,' the knowledge of his fellow-man."
*The Hospital.*

*Of all Booksellers, or of*

**The Scientific Press, Ltd.,**
**28/29 Southampton Street, Strand, London, W.C.**

